# The Effect of Emotional Arousal on Inhibition of Return Among Youth With Depressive Tendency

**DOI:** 10.3389/fpsyg.2019.01487

**Published:** 2019-07-02

**Authors:** Liwei Zhang, Huiyong Fan, Suyan Wang, Hong Li

**Affiliations:** ^1^ Research Center of Brain and Cognitive Neuroscience, Liaoning Normal University, Dalian, China; ^2^ School of Basic Medical Sciences, Jinzhou Medical University, Jinzhou, China; ^3^ College of Educational Science, Bohai University, Jinzhou, China; ^4^ College of Food Science and Engineering, Jinzhou Medical University, Jinzhou, China; ^5^ College of Psychology and Sociology, Shenzhen University, Shenzhen, China

**Keywords:** inhibition of return, depression-tendency individual, arousal, negativity bias, negative emotional face

## Abstract

The occurrence and development of depressive symptoms were thought to be closely related to excessive attention to negative information. However, the evidences among researchers were inconsistent on whether negative emotional information could induce attention bias in depressed individuals. One possible hypothesis is that the arousal level of stimuli regulates the attention bias of depressed individuals to negative emotional stimuli. In the current study, we directly assessed the attentional inhibition of depression-tendency individuals to different arousal levels of negative emotional faces. The Center for Epidemiologic Studies Depression Scale (CES-D) was used to distinguish the depression-tendency group from the health group. Thirty-three participants in each group completed a simpler cue-target task that comprised four kinds of experimental conditions, in which group was an inter-subject variable, while cue validity, arousal level, and stimulus onset asynchrony were internal variables. By subtracting the reaction time under the valid cue from the reaction time under the invalid cue, we got the magnitudes of inhibition of return (IOR), which reflected the effective suppression of previously noticed irrelevant information. We found that, in health group, the IOR effect was smaller at high arousal level than at low arousal level. This means that even in the normal population, higher arousal level of negative emotional information could weaken the individual’s attention inhibition ability. While in the depression-tendency group, the IOR effect only appeared at low arousal level condition, but in the high cue condition it showed the reversal pattern, that was, the cue effect. These results indicated for the first time that the attention bias of depressive individuals to negative emotional stimuli was influenced by the arousal level of stimuli, and the negative stimuli with high arousal level were more difficult to suppress.

## Introduction

Depression is one of the most frequent problems in human mental disorders, which seriously damages the quality of life of individuals. Although different factors may lead to the development of depressive symptoms, cognitive bias, especially attention bias and memory bias caused by negative information, play a key role (Taylor and John, 2004; Fossati, 2018). Thus, the impact of excessive attention to negative information on depressive symptoms has attracted increasing attention from researchers (Donaldson et al., 2007; Karparova et al., 2007).

On the neurological level, the over-attention to negative information in depressed individuals stems from the dysfunction of excitation and inhibition (Joormann, 2004; Joormann and Gotlib, 2007). In healthy people, the anterior cingulate gyrus regulates the processing of emotional information through functional connections with the dorsolateral prefrontal cortex. However, these regions of depressed individuals showed low participation (Rogers et al., 2004). This is thought to be related to their inability to obtain attentional suppression of negative stimuli. Researchers have confirmed this view in the inhibition of return task. Inhibition of return (IOR) is a delayed response to stimuli that appear at previously noticed locations (Posner and Cohen, 1984; Lupiáñez et al., 2006). Through this effect, researchers could directly assess the attentional inhibition of irrelevant information. In this task, the cue appears on the left or right side of the screen, followed by the target to be detected. The participants are asked to judge the position of the target. Under the valid cue condition, the target appears in the same position as the cue, while under the invalid cue condition, the target appears in the irrelevant position. In this task, IOR occurs when the response speed to the uncued target is faster than that to the cued one. Because the information that has already been noticed is automatically inhibited, this mechanism is essential to maintain people’s normal cognitive process and emotional control. Using the paradigm of IOR, Dai and Feng (2009) found that participants with severe depression showed deficient IOR for negative emotional faces. Subsequently, Baijal and Srinivasan (2011) also found that identifying sad faces produced lower amplitude of IOR than happy faces. Similar results were repeated by Perez-Duenas et al. (2014). These evidences support the previous hypothesis that attention bias to negative information is a major contributor to the development and recurrence of depressive symptoms (Taylor and John, 2004; Mathews and Macleod, 2005; Fossati, 2018). Therefore, the IOR paradigm can be used as an effective tool to effectively and quickly assess the impairment of attention control system in depressed patients or individuals with depressive tendency.

It is noteworthy that the researchers failed to obtain consistent evidence about the attentional bias of depressed individuals to negative emotional information. For example, Hill and Dutton (1989) also used negative emotional pictures, but they did not induce attention bias in depressed patients. Karparova et al. (2005) also found that in visual search task, the ability to separate attention from negative emotional faces was not impaired in depressed individuals. Some researchers tried to attribute this zero result to the inappropriate choice of experimental materials. The stimuli used by Hill and Dutton (1989) might cause anxiety in participants and therefore they were not enough to attract their attention, while the over-abstract emotional faces used by Karparova et al. (2005) might also lead to barriers at the bottom level of perception, resulting in the possibility that depressed individuals are not aware of the negative messages conveyed by these abstract emotional faces well.

However, we suspect that these researchers might overlook the fact that depressed individuals have higher overall stimulus arousal level. Unlike normal people, depressed individuals suffer from depressive rumination. Some researchers even believe that depressive rumination is an important factor in producing and maintaining depressive symptoms (Lyubomirsky et al., 2011). Specifically, depressed people tend to focus too much on their depressed moods and their meanings (Nolen-Hoeksema, 1991). This makes depressed individuals unconsciously focus on their real-time emotional state, so that they are not susceptible to external stimuli with low arousal level (Nolen-Hoeksema et al., 2008). This means that only presenting negative emotional information for depressed participants may not be enough to induce observable attention bias. Researchers also need to pay attention to the arousal level of negative emotional information. Correspondingly, emotional information was defined as a multidimensional concept, which consisted of two different dimensions: pleasure and arousal (Lang et al., 1998). The pleasure dimension, also known as valence, changes between pleasure (positive) and non-pleasure (negative); the arousal dimension changes between calm and excitement. Based on this definition, any emotion can be decomposed into these two dimensions, and different emotional experiences are only the result of a mixture of the two dimensions. However, only manipulating stimuli in terms of valence and ignoring the arousal dimension may lead to unreliable conclusions. For example, in some attention studies, researchers might compare negative high arousal with positive low arousal stimuli (e.g., Fox et al., 2001; Ohman et al., 2001). It was clearly inappropriate, especially considering the evidence that arousal level of emotional stimuli could affect the attention allocation (Vogt et al., 2008; Yang, 2018, unpublished). Similarly, in studies examining the effects of emotional information on attention bias, researchers did not pay enough attention to the two-dimensional attributes of emotional information, but differentiated emotional stimuli in the valence dimension. Therefore, the arousal level of emotional stimuli used by various research institutions might be different, resulting in inconsistent results. In the current study, we would directly assess whether depressive individuals’ attention bias to negative emotional information could be regulated by arousal dimensions.

In order to achieve this goal, we made the following settings in the experimental design: Firstly, referring to previous studies, we adopted a more sensitive inhibition of return paradigm, which was more suitable for assessing individual’s attention inhibition to negative emotional information. If depressed participants allocated excessive attention resources to negative emotional faces, they would predict deficient IOR. Secondly, we selected depression-tendency people, not depressed patients, as the research participants, which helped to test whether the finding of Dai and Feng (2009) based on depressed patients could be extended to the early stage of depressive symptoms. Finally, we distinguished negative emotional faces at two different arousal levels, which helped to directly assess the impact of different arousal levels on the inhibition of negative information. In addition, we also included healthy participants in the study, considering that no previous studies have examined the effect of arousal of emotional stimuli on IOR in healthy people. Since the arousal dimension of stimulus could affect different levels of cognitive processing (Vogt et al., 2008; Fernandes et al., 2011; Fröber and Dreisbach, 2012), we believe that for both healthy and depression-tendency participants, the arousal dimension of emotional stimulus could affect the magnitude of IOR. But the regulatory model might be different between the two groups. Specifically, our main hypothesis was that: (1) high arousal level of negative emotional stimulus reduced the IOR induced by priming stimulus; (2) depression-tendency participants with depressive tendencies have more difficulty suppressing high arousal negative information than the healthy group.

## Materials And Methods

### Participants

The present experiment included a depression-tendency group and a health group. All participants were recruited from the Jinzhou Medical University community and reported normal or corrected-to-normal vision. This experiment was approved by the Local Ethics Committee of Jinzhou Medical University with written informed consent from all participants. The recruitment procedures for participants are as follows: firstly, we used the Center for Epidemiologic Studies Depression Scale (CES-D, Radloff, 1977) to assess the degree of depression of participants, considering that it can measure multiple aspects of depressive symptomatology (depressed affect, somatic activity, interpersonal relations, and positive affect) in nonclinical settings (Radloff, 1977). The CES-D is a highly reliable and valid instrument that has been widely used to screen depression in adults (Mui et al., 2001; Long et al., 2002; Spijker et al., 2004; Yang et al., 2009). It has been evaluated as having good nomological validity (Karim et al., 2015) with total score showing high internal consistency coefficients (*α* = 0.85 to *α* = 0.90) (Santor et al., 2006; Wei et al., 2010). Correlation coefficient of the CES-D scores with other main depression scales has ranged from 0.81 to 0.91 (Weissman et al., 1977; Tatar et al., 2013). The CES-D is a 4 item Likert-type scale scoring from 0 (less than 1 day per week) to 3 (5–7 days a week) on 20 items with total score was between 0 and 60. The higher score indicated higher depression. Previous studies have pointed out that individuals with a total score above 16 can be considered to have a clinically relevant level of depressive symptoms (Radloff, 1977; Sonnenberg et al., 2013). According to this criterion, we adopt a more conservative strategy, that is, the participants with scores higher than 25 were assigned to the depression-tendency group, and the participants with scores lower than 5 were included in the health group. Based on the effect size obtained from previous relevant studies (Dai and Feng, 2009), a minimum of 30 participants in each group would be necessary to achieve 80% power. Accordingly, 33 participants were selected from each group, with 15 males in the health group and 16 males in the depression-tendency group. All participants were undergraduates with age ranging from 19 to 22 years, and they reported no history of depressive disorders and other psychological disorders. As shown in Table 1, *t*-test results revealed no difference in age (*t*(64) = −1.09, *p* = 0.28), but the difference in depression score was significant across the two groups (*t*(64) = −30.36, *p* < 0.001).

**Table 1:** Basic information of participants (M ± SD).

Groups	Sample size	Age	Depression score
Normal control	33	19.18 ± 3.63	0.88 ± 0.86
Depressive-tendency	33	20.42 ± 5.39	32.73 ± 5.97
		*t* = −1.09	*t* = −30.36**

** *p < 0.01*.

### Stimuli

Because, compared with words, the facial pictures contain more social and interpersonal information which can effectively induce individual emotional experiences (Karparova et al., 2005; Mogg and Bmdley, 2005), the present paper adopted face pictures of young children developed by Luo and his colleagues (Luo et al., 2015). All the face pictures were designed in the same size (about 260 × 300 pixels and 9 cm × 10 cm) (Goeleven et al., 2006).

Other 60 participants were asked to assess the pleasure and arousal of those pictures on a scale of 1–9, and the results recorded the consistency of the scores. Sad face pictures that scored over 4 with 70% consistency or more were chosen as experimental stimuli (Eizenman et al., 2003; Koster et al., 2006). Finally, the present experiment used 18 face pictures with high arousal and 18 pictures with low sadness arousal, averaging 4.95 and 6.60, respectively.

### Design

Previous studies tended to use 250, 500, and 750 ms as SOA between the cue and targets. Interestingly, some studies have found that depressive individuals exhibited IOR under the condition of stimulus onset asynchrony (SOA) at 14 ms (Dai and Feng, 2009). To allow enough time for subjects to respond, SOA in this study was divided into three categories: 14, 250, and 750 ms.

We used a mixed design, with groupings (normal and depression tendency), SOAs (14, 250, and 750 ms), Cue validity (validity and invalidity) and levels of arousal (high and low) were adopted as variables in our study (Koster et al., 2006).

About the mixed design, the grouping was a between-subjects variable, and the other three variables were within-subjects factors. By subtracting the reaction time under the valid cue from the reaction time under the invalid cue, we could get the magnitude of IOR, in which the positive value indicated the existence of IOR, while the negative value was called as the cue effect (Dai and Feng, 2009).

Each participant completed 216 trials in total including 108 cue-validity trials (i.e., the target appeared in the same location as the cue) and 108 cue-invalidity trials (i.e., the target appeared in the other location to the cue). The cue was presented for 1,000 ms (Waters et al., 2007).

### Procedure

Appointments about the time, places, and contents of the experiment were made with the participants by telephone. After arriving at the laboratory, participants were informed of the experimental process again and were given written informed consent. They had the right to withdraw at any stage during the experiment. The subjects took a 10-min break to become familiar with the laboratory environment.

E-prime 2.0 (developed by Carnegie Mellon University and Pittsburgh University) was used to present the stimuli, record response time and accuracy. The screen on which two boxes 9 cm × 10 cm in size with white edges were 17 cm apart from each other (calculated the distance of two centers) was on a black background. Black-white face pictures were presented on either of the two boxes randomly (Koster et al., 2006).

Once arriving at the laboratory, participants were asked to read the instructions first as follows: “Look at the + presented in the center of the screen. There are two boxes on the left and right sides on the screen, and one face picture would appear on either the left-side box or the right-side box. Then, # is presented on the same or different side of the face after the face picture disappears. Please press the **F** key immediately when the # sign appears on the left side, and press the **J** key when the # sign appears on the right side. Please focus on the center of the screen and respond as quickly and as accurately as possible.”

**Figure 1:**
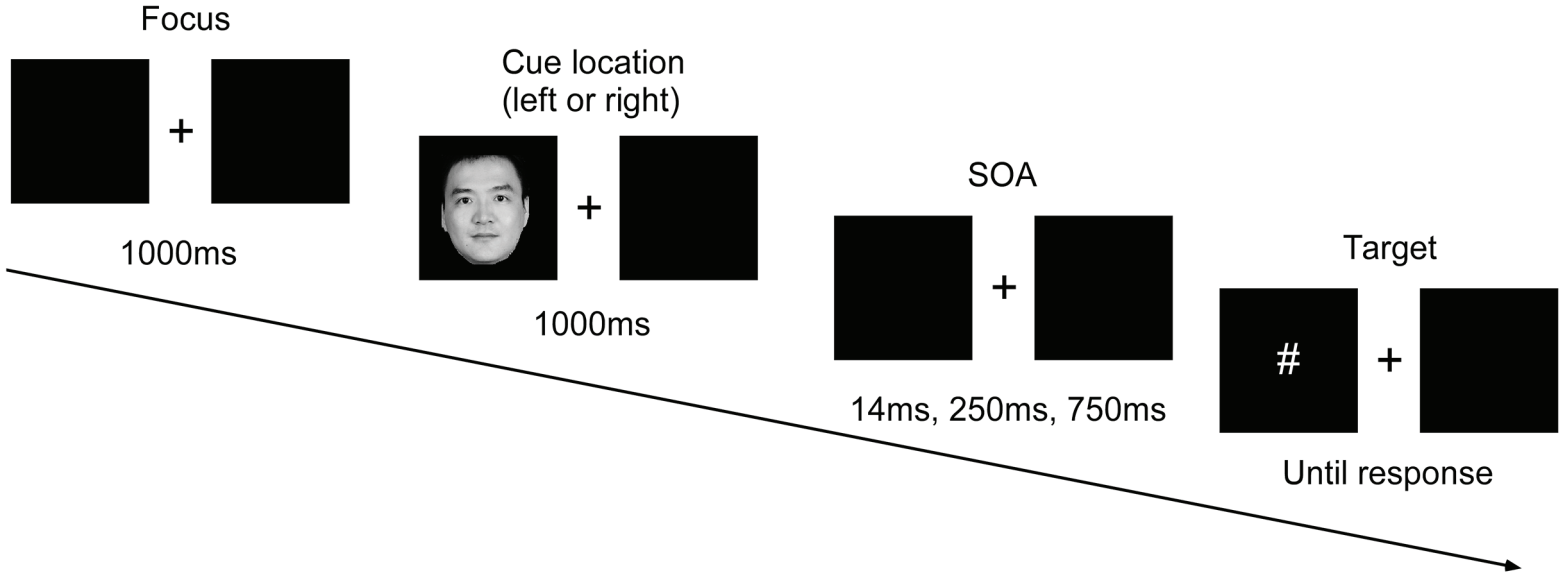
Sequence of events in a single trial. SOA, stimuli onset asynchrony. Written informed consent was obtained from the depicted individual for the publication of his identifiable image.

Participants firstly carried out practice trials (in the presence of experimenter), then entered the experiment (in the absence of experimenter) until they mastered the tasks completely. Throughout the experiment, participants were seated 60 cm away from the computer monitor, with their index fingers on the F and J keys on the keyboard. Faces appeared randomly on the left or right side of the box. Figure 1 shows a full trial of the experiment.

## Results

**Figure 2:**
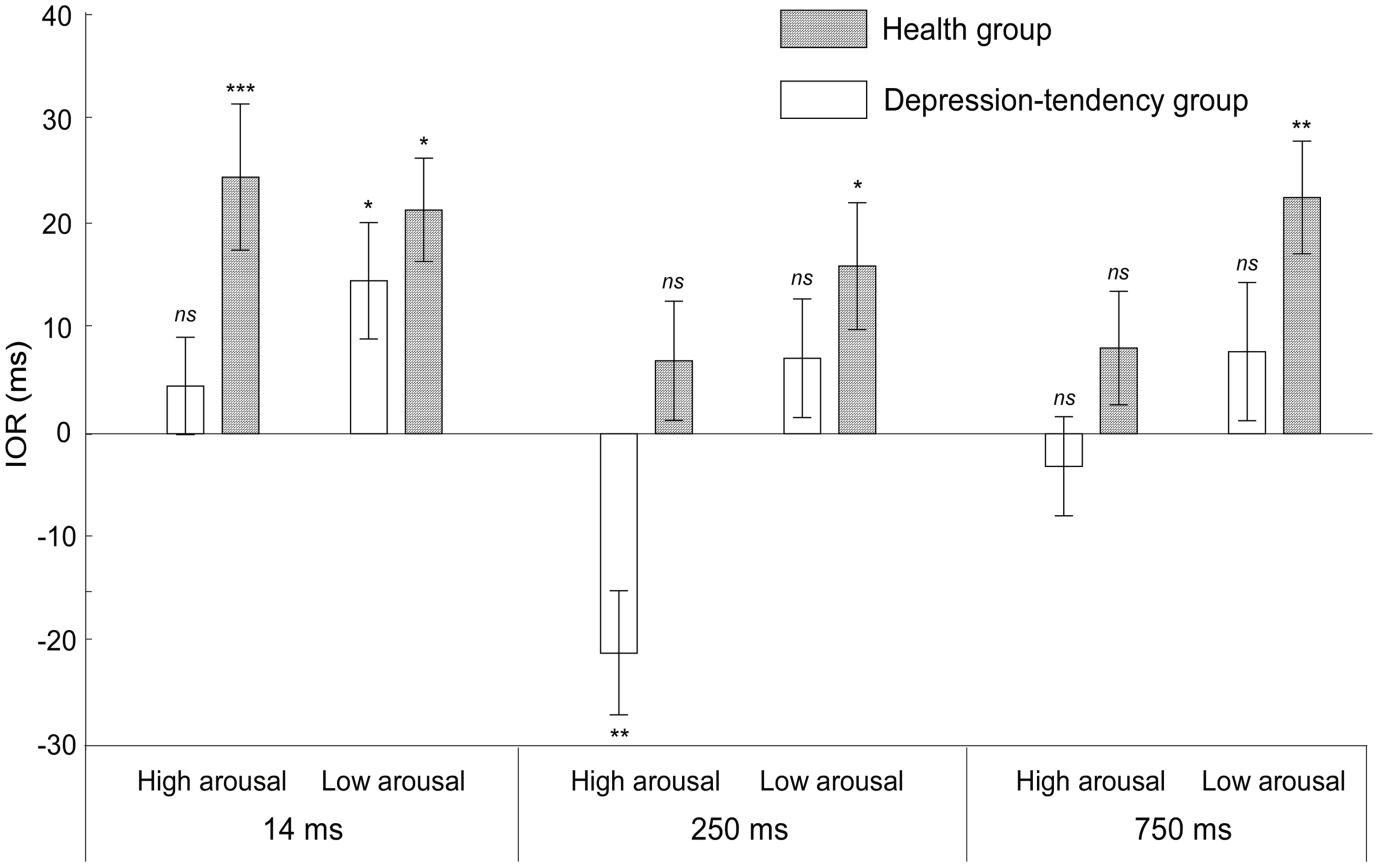
The inhibition of return (IOR) values of the depression-tendency group and the health group were measured at different SOAs (14, 250, and 750 ms) and two arousal levels (high arousal and low arousal). IORs were calculated by subtracting the RT under the valid cue from the RT under the invalid cue. The interval *p* of the *t-*test between valid cue and invalid cue was marked. ns, non-significant. *p < 0.05, **p < 0.01, and ***p < 0.001.

Under all conditions, participants could perform tasks with high accuracy. Referring to previous studies, we mainly focus on their performance in Response time (RT). Incorrect-response data were excluded before the start of data analyses (Gouzoulis-Mayfrank et al., 2006). The results are presented in Table 2 and Figure 2.

**Table 2:** Response time under different conditions (M ± SD, ms).

		Health group(SOAs)			Depressive-tendency group(SOAs)		
Arousal	Cue validity	14 ms	250 ms	750 ms	14 ms	250 ms	750 ms
	Valid	420 ± 59	395 ± 61	367 ± 44	451 ± 82	405 ± 73	387 ± 72
High	Invalid	396 ± 52	388 ± 57	359 ± 55	447 ± 86	426 ± 78	390 ± 77
	IOR	25 ± 40	7 ± 33	8 ± 31	5 ± 27	−21 ± 34	−3 ± 27
	Valid	421 ± 52	397 ± 54	377 ± 52	454 ± 80	420 ± 73	402 ± 78
Low	Invalid	400 ± 51	381 ± 54	354 ± 49	440 ± 79	412 ± 71	394 ± 81
	IOR	21 ± 28	16 ± 35	23 ± 31	15 ± 32	7 ± 33	8 ± 34

According to the hypothesis which is put forward in the introduction, we mainly focus on whether different arousal levels of negative emotional faces (high arousal vs. low arousal) could affect their IOR differently in two groups. For this purpose, we performed a mixed 4-factor ANOVA, and group (health group vs. depression-tendency group) was an inter-subject variable, while cue validity (valid cue vs. invalid cue), arousal levels (high arousal vs. low arousal) and SOAs (14, 250, 750 ms) were internal variables. The results showed a significant main effects of SOA (*F*(2, 128) = 152.69, *p* < 0.001, $\eta_{p}^{2}$ = 0.71), in which the longest response time was when SOA was 14 ms. In addition, there is a significant main effects of cue validity (*F*(1, 64) = 14.35, *p* < 0.001, $\eta_{p}^{2}$ = 0.30), with shorter response time under invalid cue, and a significant three-factor interaction with the variables of arousal level, cue validity and grouping (*F*(2, 128) = 4.62, *p* < 0.05, $\eta_{p}^{2}$ = 0.08).

In order to further illustrate the interaction of the three factors, two-factor ANOVA of 2 (arousal levels) × 2 (cue validity) under different groups was carried out. In the health group, we found that the main effect of cue validity was significant (*F*(1, 32) = 91.75, *p* < 0.05, $\eta_{p}^{2}$ = 0.74), in which the RT of invalid cues was shorter than that of valid cue; the interaction between arousal level and cue validity was significant (*F*(1, 32) = 5.61, *p* < 0.05, $\eta_{p}^{2}$ = 0.15). This interaction effect showed that different arousal levels could affect cue validity. In order to show more details about this effect on the IOR, we next performed the simple effect analysis. By subtracting the RT under the valid cue from the RT under the invalid cue, we could get the magnitude of IOR, in which the positive value indicated the existence of IOR, while the negative value was the reverse mode of IOR, i.e., the emergence of cue effect. We found that the IOR effect was smaller at high arousal level than at low arousal level (*t*(32) = −2.36, *p* < 0.05, *Cohen’s d* = 0.442, different IOR = −7 ms). This means that even in the healthy population, higher arousal level of negative emotional information could weaken the individual’s attention inhibition ability.

While in the depression-tendency group, we found that only the interaction between arousal level and cue validity was significant, (*F*(1, 32) = 22.63, *p* < 0.001, $\eta_{p}^{2}$ = 0.41), further simple effect analysis showed that the IOR showed a completely opposite pattern at different arousal levels. At low arousal level, RT under valid cue was longer than that under invalid cue (*t*(32) = 2.629, *p* < 0.05, *Cohen’s d* = 0.447, IOR = 10 ms), i.e., showed an IOR effect. However, at high arousal level, RT under valid cue was shorter than that under invalid cue (*t*(32) = −2.029, *p =* 0.051, *Cohen’s d* = 0.348, IOR = −7 ms), i.e., showed cue effect. These results strongly supported our hypothesis that depression-tendency individuals’ ability to suppress negative emotional stimuli was regulated by the arousal dimension of stimuli, and they could not effectively inhibit the attraction of high arousal negative emotional faces, that is, inevitable attentional bias to these information. Details of IOR under all conditions are shown in Figure 2.

## Discussion

Current studies have focused on whether high arousal levels of negative emotional information could worsen the attention inhibition ability of depression-tendency individuals. We used the inhibition of return paradigm to assess participants’ inhibition of the spatial location of previously noticed stimuli. This ability helps them to automatically orient their attention to the target location in complex environments, thus supporting the normal functioning of the cognitive system. The results were concise and interesting: We found that negative emotional faces at high arousal levels reduced the IOR induced by priming stimuli compared with those at low arousal levels, which was observed in both healthy and depression-tendency participants. The key evidence was that depression-tendency individuals suffered more from attentional bias when confronted with high arousal levels of negative emotional stimuli than healthy participants.

Our evidence replicated and extended previous findings. Similarly, using the inhibition of return paradigm, Dai and Feng (2009) confirmed for the first time that people with severe depression exhibited a marked absence of attentional inhibition on negative emotional faces. They argued that the lack of inhibition of negative information made it difficult for depressed patients to resist the interference of negative events. This allowed them to experience more depression and to lead to the persistence and deterioration of depressive symptoms. Consistent with their findings, our evidence suggested that depression-tendency individuals exhibited a markedly absent inhibition on negative emotional faces, and this effect was observed in different stimulus onset asynchrony. However, our evidence extended the study of Dai and Feng (2009) in three ways. First, we demonstrated that insufficient suppression of negative emotional information has already been observed in depression-tendency individuals who were not enough to be diagnosed with depression. This finding makes sense. Combining the evidence of Dai and Feng (2009), it means that the deficient attentional inhibition of negative information has emerged early in the progression of depressive symptoms. In fact, researchers have observed attentional bias to negative stimuli in individuals with potential depressive symptoms. For example, young people whose mothers had a history of depression were more likely to show attention bias to negative stimuli (Joormann et al., 2007). In addition, when showing self-related emotional vocabulary, twins with family history of depression showed significant over-attention to negative information (Miskowiak et al., 2015). However, these studies could not distinguish whether attention bias was caused by facilitation mechanism or inhibition mechanism. Using the paradigm of inhibition of return, our evidence tended to support that the attention bias of depression-tendency individuals toward negative stimuli might arise from their failure of attention inhibition toward negative stimuli.

More importantly, the evidence we provide showed for the first time that the arousal dimension of stimulus could significantly affect the attentional inhibition of negative emotional information in depression-tendency individuals. In other words, they did not treat all negative information equally, but they tended to pay excessive attention to negative stimuli of high arousal levels. This finding can be explained by parallel distributed processing model. This model assumed that individuals have multiple different inputs to the nervous system (Cohen et al., 1990). For depressed population, the threshold of neuron population for processing general external information is higher. This leads them to pay too much attention to their inner feelings and to reduce their sensitivity to external stimuli. However, some of their other input neurons have lower thresholds, which are responsible for processing depression-related information. This is due to the strong activity of neurotransmitters related to depression, which leads to the higher activation level of nerve pathways to depression-related stimuli. Therefore, when negative stimuli with higher arousal levels were given to depressed individuals, the information was more easily received by the input units of processing those stimuli. Consistently, we found that even for negative emotional information at the same arousal level, depression-tendency participants tended to exhibit lower or even disappeared IOR than healthy people. This may be due to the low activation threshold of neurons processing negative stimuli.

However, it should be pointed out that the current research also has some limitations. First, our evidence did not allow us to distinguish whether impaired attention suppression is a cognitive factor that contributes to the development of depressive symptoms or the cognitive characteristics associated with the development of depressive symptoms. But combined with other research evidence, the former seems more reasonable. There was already a view that cognitive bias toward negative information is an important cause of depressive symptoms (Taylor and John, 2004). Consistent with this view, studies have shown that attention bias to negative stimuli was associated with depression susceptibility (MacLeod et al., 2002). In addition, depressed patients at different stages all have attention bias to negative stimuli (Nandrino et al., 2004). Secondly, we only selected negative emotional faces as experimental materials, without considering the possible interaction between arousal dimension and valence dimension of emotional stimuli. Taking into account previous research findings, attention bias can be driven by the arousal dimension of stimulus rather than its valence dimension (Schimmack and Derryberry, 2005), future studies need examine whether depressed individuals have the same deficient attention inhibition to other high arousal levels of information, such as anxiety-related scenarios.

Overall, the evidence provided by this study suggested that the arousal of stimulus could regulate individuals’ excessive attention to negative information. In addition, the ability of depression-tendency individuals to inhibit irrelevant negative information has already been impaired.

## Ethics Statement

The study was approved by the Local Ethics Committee of Jinzhou Medical University.

## Author Contributions

LZ, SW, and HF conceived and designed the experiment. LZ, HF, and SW performed the experiment and analyzed experimental data. LZ, SW, HF, and HL wrote the manuscript. LZ and HF contributed equally to this work.

### Conflict of Interest Statement

The authors declare that the research was conducted in the absence of any commercial or financial relationships that could be construed as a potential conflict of interest.
